# Three Cases of Transtracheal Catheter Oxygenation for Postoperative Dyspnoea with Pituitary-Dependent Hyperadrenocorticism in Dogs Treated by Surgery

**DOI:** 10.1155/2022/7389661

**Published:** 2022-03-22

**Authors:** Sachiyo Tanaka, Shuji Suzuki, Takahiro Teshima, Hirokazu Ishino, Asaka Sato, Nobuo Kanno, Yasushi Hara

**Affiliations:** ^1^Faculty of Veterinary Science, Laboratory of Veterinary Surgery, Nippon Veterinary and Life Science University, Tokyo, Japan; ^2^Faculty of Veterinary Science, Laboratory of Veterinary Internal Medicine, Nippon Veterinary and Life Science University, Tokyo, Japan; ^3^Faculty of Veterinary Science, Laboratory of Small Animal Surgery, School of Veterinary Medicine, Kitasato University, Aomori, Japan; ^4^Faculty of Veterinary Science, Azabu University Veterinary Teaching Hospital, Kanagawa, Japan

## Abstract

Transsphenoidal surgery (TSS) is a curative treatment for pituitary-dependent hyperadrenocorticism, and its use in dogs has recently increased. One of the most serious postoperative complications of TSS is dyspnoea. We report three cases where transtracheal catheter oxygen therapy prevented death from respiratory distress secondary to enlarged soft palate after TSS.

## 1. Introduction

Cushing's syndrome (CS) is a major endocrine disorder in dogs, with a reported annual incidence of 1–2 cases per 1,000 dogs [1]. Pituitary-dependent hyperadrenocorticism (PDH) caused by pituitary corticotroph adenoma (PCA) is the most common cause of CS, accounting for approximately 80–85% of cases [1, 2]. In veterinary medicine, transsphenoidal surgery (TSS) is recommended as the first-line treatment for pituitary adenomas without enlargement [3, 4].

The favourable outcome of TSS has resulted in its increased application [5–7]. However, fatal postoperative complications have been reported within 4 weeks of surgery, including prolonged coma, intraoperative arterial haemorrhage, hypernatraemia, and dyspnoea [5, 8–10]. It has been reported that dyspnoea is one of the most common fatal postoperative complications, accounting for 16.67% of all postoperative deaths within 4 weeks [8]. In TSS, incision and suturing of the soft palate might cause inflammation-induced swelling postoperatively, resulting in upper airway obstruction syndrome [3]. Low-flow oxygen delivery using a transtracheal catheter (TTC) enables safe and effective oxygenation and ventilation of dogs and cats with upper airway obstruction [11, 12]. Herein, we describe three cases of dyspnoea caused by an enlarged soft palate after TSS and improvement of respiratory status with TTC-oxygen therapy.

## 2. Case Presentation

### 2.1. Case 1

**Figure 1:**
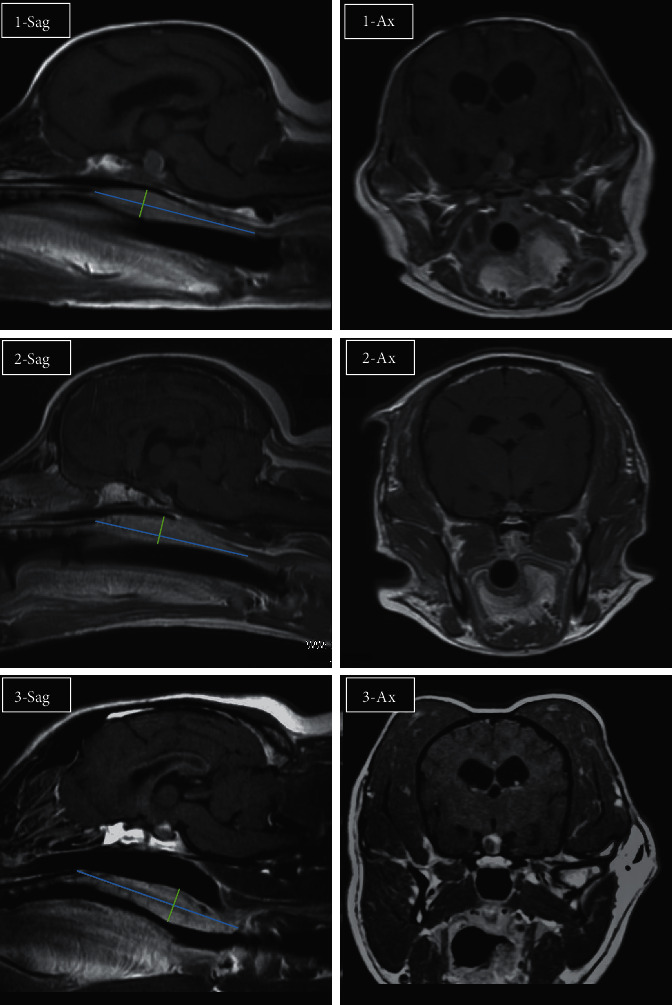
MRI images of the pituitary gland and soft palate of the three dogs in this study. 1-Sag, 2-Sag, and 3-Sag: gadolinium-enhanced T1-weighted images of middle-sagittal section in cases 1, 2, and 3, respectively. 1-Ax, 2-Ax, and 3-Ax: gadolinium-enhanced T1-weighted image of the middle-axial section in cases 1, 2, and 3, respectively. The cranial MRI scans were performed using a 1.5 T superconducting MR imaging system (Visart; Toshiba, Tokyo, Japan) or a 3.0 T superconducting MR imaging system (Signa HDxt; GE Healthcare, Tokyo, Japan) under the following conditions: slice thickness of 2 mm with no slice gap, matrix of 160 × 256, and field of view of 12 cm (1.5 T MRI) or slice thickness of 2 mm with no slice gap, matrix of 320 × 256, and field of view of 15 cm (3.0 T MRI). T1- and T2-weighted images were taken with a repetition time/echo time of 410/15 ms and 4,000/100 ms, respectively. The length of the soft palate was measured as a straight line from the end of the hard palate to the caudal end of the soft palate in the middle-sagittal section (blue lines). The thickness of the soft palate was measured perpendicular to this line, and the maximum value was recorded (green lines). MRI: magnetic resonance imaging.

**Figure 2:**
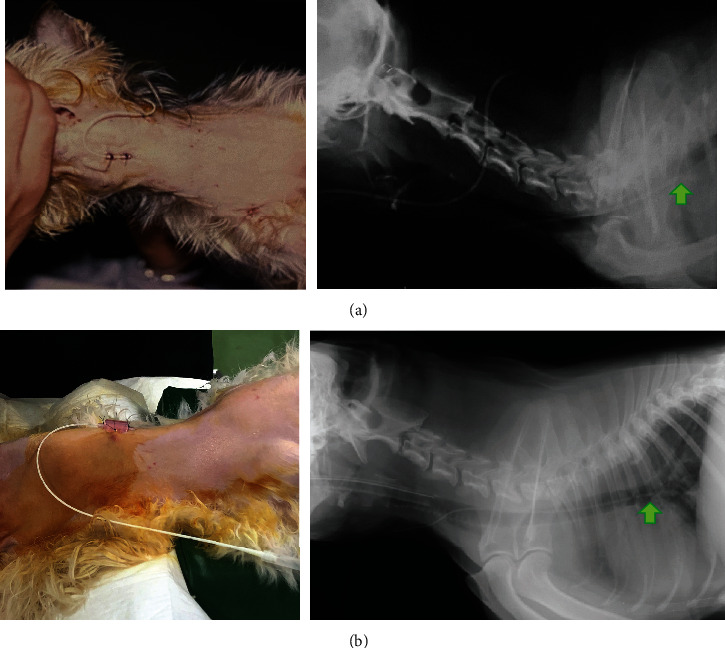
Photographs and X-ray images of cases 1 and 2: (a) photographic images of the neck showing transtracheal catheter placement after transsphenoidal surgery and (b) X-ray images of the neck taken to check the placement of the transtracheal catheter. Arrows in the X-ray images indicate the apex of the catheter in the thoracic cavity.

An 11-year-old spayed female Yorkshire terrier weighing 3.2 kg presented to the Department of Pituitary Surgery at our institution. The dog had been examined by a home doctor (HD) for clinical signs of polyuria and polydipsia (PU/PD) 1 month prior to its presentation. An adrenocorticotropic hormone (ACTH) stimulation test showed endogenous and poststimulation cortisol concentration (ECC and PCC) of 11.0 pg/mL and 31.4 pg/mL, respectively. The dog was suspected to have hyperadrenocorticism (HAC) and was referred to our institution for further management. On arrival, the dog was panting, but with no breath sounds or snoring. Abdominal ultrasonography showed an adrenal dorsoventral thickness (ADT) [13, 14] of 6.5 mm each for the left adrenal gland (LAG) and right adrenal gland (RAG). During tracheal intubation for magnetic resonance imaging (MRI), an elongated soft palate was visually confirmed. The displacement of posterior pituitary on MRI [15], raised pituitary-brain ratio (PBR) of 0.43 [16], and MRI-based classification of grade III [17] confirmed the diagnosis of PDH. The ratio of the maximum thickness of soft palate (SPT)/body weight (BW, cm/kg) was 0.21 [18] (Figure 1). One month of treatment with mitotane (10 mg/kg, SID) showed no improvement in PU/PD, and the dog developed gastrointestinal symptoms and loss of appetite. Mitotane was discontinued. Two months after the initial visit, examination of the respiratory system and chest radiography revealed no abnormalities, and TSS was performed according to the technique reported by Meij et al. [3]. At 15 hours postoperatively, the dog developed respiratory distress despite being on supplemental oxygen (fraction of inspiratory oxygen (FiO_2_): 40%). A central venous catheter kit (CV Regaforce DX, TERUMO CORPORATION, Tokyo, Japan) was inserted through the midline of the neck into the trachea. The position of the catheter tip anterior to the intrathoracic tracheal bifurcation through the anterior thoracic foramen was confirmed by X-ray (Figure 2) [19, 20]. TTC-oxygen therapy was administered at a flow rate of <50 mL/kg/min [19]; the respiratory status improved rapidly, and saturation of percutaneous oxygen (SpO_2_) remained consistently above 97%. After confirming that SpO_2_ could be maintained above 97% at an FiO_2_ of 20%, the TTC was removed 4 days after surgery. The dog was discharged 10 days after the surgery.

### 2.2. Case 2

A 5-year-old, spayed female, mixed-breed dog weighing 5.65 kg was referred to our institution after presenting to an HD with clinical signs of PU/PD for 13 months. The ECC was 4.5 pg/mL, and PCC was 38.5 pg/mL. Abdominal ultrasonography was highly suggestive of ADT, with the LAG and RAG measuring 5.3 mm and 6.2 mm, respectively [13, 14]. The dog had been treated with trilostane (0.39–1.56 mg/kg, BID) for 7 months before being referred to our institution. However, PU/PD persisted and activity disappeared. On arrival, the dog was panting, but with no breath sounds or snoring. Abdominal ultrasonography showed an ADT of 10.6 mm for LAG and 9.7 mm for RAG. During tracheal intubation for MRI, an elongated soft palate was visually confirmed. The displacement of posterior pituitary on MRI [15], PBR of 0.18 [16], and an MRI-based classification of grade II [17] confirmed the diagnosis of PDH. SPT/BW was 0.14 (Figure 1). At the owner's request, TSS was performed 1 month after the first visit. Approximately 7 hours postoperatively, the dog developed respiratory distress while on 40% FiO_2_ supplementation. A TTC was placed in the trachea (Figure 2), and oxygen therapy was initiated [19]; the respiratory status improved rapidly and SpO_2_ remained consistently above 97%. TTC was used for 1 day during which the oxygen requirement reduced to 20% FiO_2_. Approximately 40 hours after the surgery, the dog developed acute hypernatraemia (171 mmol/L), which resulted in persistent seizures and was managed under ventilation. The dog was euthanised 10 days after the operation due to hypernatraemia-induced brainstem damage.

### 2.3. Case 3

A 6-year-old male Beagle dog weighing 12.8 kg was referred to our institution after presenting to an HD with clinical signs of PU/PD for 4 months. On arrival, the dog was panting and snoring, with no breath sounds. ACTH stimulation test was suggestive of HAC (ECC: 0.2 pg/mL, PCC: 40.7 pg/mL), and the dog was treated with trilostane (3–4 mg/kg, SID) for 1 month. However, PU/PD persisted. The ADT on abdominal ultrasonography was 7.0 mm each for LAG and RAG [13, 14]. Elongation of the soft palate was observed during tracheal intubation for MRI. Enlarged and displaced posterior pituitary on MRI (PBR: 0.47) [15, 16] and an MRI-based classification of grade III [17] confirmed the diagnosis of PDH. SPT/BW was 0.12 [18] (Figure 1). At the owner's request, TSS was performed 1 month later. Respiratory distress developed 18 hours postoperatively while on 40% FiO_2_ supplementation; a TTC was placed in the trachea and oxygen therapy was initiated. The respiratory status improved rapidly and SpO_2_ remained consistently above 97%. After confirming that SpO_2_ could be maintained above 97% at 20% FiO_2_, the TTC was removed 3 days after surgery. The dog was discharged 11 days after the operation.

## 3. Discussion

In all three cases, respiratory distress caused by an enlarged soft palate occurred 7–18 hours after surgery, and mortality from dyspnoea was averted with the use of TTC-oxygen therapy. Therefore, TTC-oxygen therapy may be useful for ventilatory impairment associated with temporary postoperative swelling of the soft palate. To our knowledge, this is the first report on the successful use of TTC-oxygen therapy for postoperative dyspnoea.

For healthy awake dogs, low-flow oxygen delivery via TTC < 50 mL/kg/min achieves an inspiratory oxygen concentration of approximately 50%, and at 10 mL/kg/min, a haemoglobin oxygen saturation of 97% can be maintained [19]. In the present study, TTC-oxygen therapy of ≤50 mL/kg/min was used and the SpO_2_ was persistently >97%. Better oxygenation is achieved with TTC-oxygen therapy, as compared to oxygen cages, due to airway pressure-ensured lung capacity and improved ventilatory efficiency [21]. However, care should be taken when using high-flow oxygen ventilation as it can increase airway pressure excessively and cause overinflation of the lungs [20, 22].

In the present study, all three patients had elongated soft palate, with an SPT/BW ratio of 0.12–0.21. Recently, thickening of the soft palate has been reported to be the most important predictor of severity of short airway syndrome, with SPT/BW values of 0.10 ± 0.03 and 0.15 ± 0.04 predicting nonshort airway and short airway syndromes [18]. The three dogs in this study had thick soft palates preoperatively, suggesting that placement of TTC during TSS in dogs with elongated or thickened soft palates may be useful in postoperative respiratory management. In conclusion, averted mortality from dyspnoea in all three patients with the use of TTC-oxygen therapy highlights its importance in the management of temporary airway narrowing and dyspnoea after TSS.

## Data Availability

All data relevant to the study are included in this article.
